# Airway smooth muscle function in asthma

**DOI:** 10.3389/fphys.2022.993406

**Published:** 2022-10-05

**Authors:** Dora (Jun Ping) Xiong, James G. Martin, Anne-Marie Lauzon

**Affiliations:** ^1^ Meakins-Christie Laboratories, Research Institute of the McGill University Health Centre, Montreal, QC, Canada; ^2^ Department of Medicine, McGill University, Montreal, QC, Canada

**Keywords:** airway smooth muscle, airway hyperresponsiveness, asthma, airway smooth muscle contraction, airway remodeling

## Abstract

Known to have affected around 340 million people across the world in 2018, asthma is a prevalent chronic inflammatory disease of the airways. The symptoms such as wheezing, dyspnea, chest tightness, and cough reflect episodes of reversible airway obstruction. Asthma is a heterogeneous disease that varies in clinical presentation, severity, and pathobiology, but consistently features airway hyperresponsiveness (AHR)—excessive airway narrowing due to an exaggerated response of the airways to various stimuli. Airway smooth muscle (ASM) is the major effector of exaggerated airway narrowing and AHR and many factors may contribute to its altered function in asthma. These include genetic predispositions, early life exposure to viruses, pollutants and allergens that lead to chronic exposure to inflammatory cells and mediators, altered innervation, airway structural cell remodeling, and airway mechanical stress. Early studies aiming to address the dysfunctional nature of ASM in the etiology and pathogenesis of asthma have been inconclusive due to the methodological limitations in assessing the intrapulmonary airways, the site of asthma. The study of the trachealis, although convenient, has been misleading as it has shown no alterations in asthma and it is not as exposed to inflammatory cells as intrapulmonary ASM. Furthermore, the cartilage rings offer protection against stress and strain of repeated contractions. More recent strategies that allow for the isolation of viable intrapulmonary ASM tissue reveal significant mechanical differences between asthmatic and non-asthmatic tissues. This review will thus summarize the latest techniques used to study ASM mechanics within its environment and in isolation, identify the potential causes of the discrepancy between the ASM of the extra- and intrapulmonary airways, and address future directions that may lead to an improved understanding of ASM hypercontractility in asthma.

## Asthma

According to the 2018 Global Asthma Report, 339 million people are affected by asthma worldwide, leading to 1,000 deaths daily (Network, 2018). Asthma is an inflammatory disease characterized by reversible airway obstruction and airway hyperresponsiveness (AHR), an excessive narrowing of the airways in response to various stimuli. Bronchospasm in asthma has conventionally been alleviated with bronchodilators [β_2_-adrenergic receptor agonists that relax airway smooth muscle (ASM)] underscoring the significant role played by ASM in bronchoconstriction. However, in more severe cases anti-inflammatory drugs such as leukotriene modifiers (Patel and Shaw, 2015; Wang et al., 2015) and oral corticosteroids (Sy and Siracusa, 2016) are also used, as well as the newer agents that target more specific inflammatory pathways, namely antibodies against interleukin (IL)-5, IL-5 receptor α receptor, IL-13, IL-4 receptor α, IgE, the alarmins [thymic stromal lymphopoietin (TSLP), IL-25 and IL-33], and chemoattractant receptor-homologous molecule expressed on T helper type 2 cells (CRTH2) antagonists (Zhu et al., 2018). While the inflammatory cells and mediators create an environment with conditions for exacerbations, the exaggerated contraction of the ASM is the ultimate culprit of airway obstruction and AHR. Thus, is the ASM altered genetically in asthma, or is it altered due to allergenic, viral or pollution exposures, and is mechanical stress positively feeding more mechanical alterations? These are all questions that remain only partly answered.

## Airway hyperresponsiveness in asthma

AHR is defined as an increase in airway narrowing in response to various pharmacological, chemical, and physical stimuli that would otherwise not induce any effect in healthy subjects (Baroffio et al., 2009). The stimuli can be described either as direct or indirect, with direct stimuli acting on specific ASM receptors (e.g., acetylcholine, histamine, cysteinyl leukotrienes, and prostaglandins), or indirect stimuli that act through various intermediate pathways that often involve mediators released from inflammatory cells (e.g., exercise, hypertonic aerosols, and adenosine) (Cockcroft, 2010).

The severity of AHR correlates with the severity of asthma, thus measurements of airway responsiveness have been deemed useful for confirming the diagnosis of asthma and potentially for determining the intensity of treatment required to control symptoms (Juniper et al., 1981; O’Byrne and Inman, 2003). Airway challenge tests using inhaled histamine or methacholine are commonly used and their results are quantified using the volume of air exhaled within the first second of a forced expiration (FEV_1_). In this measurement, increasing doses of contractile agonists are administered to subjects until a 20% decrease in FEV_1_ is reached (Chai et al., 1975; Juniper et al., 1981; Cockcroft, 2010). The concentration used to attain this drop is defined as the provocative concentration (PC_20_) (Cockcroft, 2010). This value is generally lower in asthmatics (PC_20_ < 8 mg/ml) than non-asthmatics (PC_20_ > 16 ml), thus a lower PC_20_ is indicative of greater airway responsiveness (O’Byrne and Inman, 2003). Dosimeters are also employed and equivalence between the provocative doses and concentrations has been determined (Coates et al., 2017).

**FIGURE 1:**
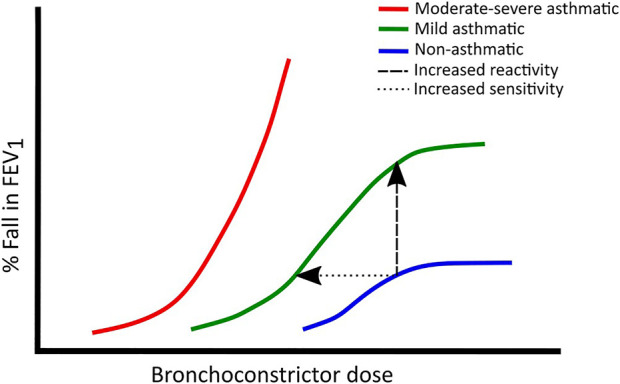
Typical dose-response curves in non-asthmatics (blue), mild asthmatics (green) and moderate to severe asthmatics (red). Leftward shifts show increased sensitivity whereas upward shifts show increased reactivity. Adapted from (Woolcock et al., 1984).

It is also possible to assess airway responsiveness by comparing agonist dose-response curves of asthmatics and non-asthmatics. *In vitro* ASM strips are known to produce a sigmoidal dose-response curve when exposed to different concentrations of histamine or methacholine (Brink et al., 1980). A similar curve can also be fitted to FEV_1_ responses of subjects exposed to inhaled agonists at various concentrations, spanning a non-response inducing concentration, through a quasi-linear increase, to a plateau where no further fall in the FEV_1_ is evoked (Woolcock et al., 1984). The position and slope of this curve can be used as an indication of the sensitivity and reactivity of the airways, respectively. Hyperresponsive airways are more sensitive and reactive to stimuli, thus asthmatic subjects tend to produce curves that are steeper and shifted leftwards compared to non-asthmatics (Woolcock et al., 1984). In only the mildest asthmatics is it possible to demonstrate a limit to the bronchoconstrictive response with increasing doses of agonist (Woolcock et al., 1984) (Figure 1).

Nowadays, mechanical properties of the respiratory system can be measured by forced oscillometry (Dubois et al., 1955; Ram et al., 2019; King et al., 2020). Perturbations are applied during tidal breathing and thus the technique has the advantage of being non-invasive, versatile, and requiring minimal patient cooperation (Kim et al., 2001; Oostveen et al., 2003). Measurements are usually carried out with a stimulus being applied to the respiratory system through the mouth. The patient breathes through a mouthpiece while supporting their cheeks with their hands and blocking the nose with a noseclip. While breathing, a sound generator applies air flow oscillations of varying frequencies to the airstream and respiratory tract and the resulting pressure is measured (Ram et al., 2019). Using Fourier transform techniques, complex impedance is calculated which can be resolved into its resistive and reactive (elastic and inertial) components (Bates, 2009). The oscillations at low frequencies (<2 Hz) give information predominantly about the rheological properties of the lung tissues whereas the higher frequencies (4 Hz > f < 32 Hz) estimate mostly the properties of the proximal and distal airways (Bates, 2009; Ram et al., 2019). While these techniques have been very useful in clinical settings to evaluate patient’s asthma, they only provide indirect information about ASM mechanics.

It has long been hypothesized that during the development of asthma the ASM undergoes fundamental changes that make it hypercontractile. Bronchoprovocation studies using a wide variety of stimuli have shown that AHR is not specific to one agonist, indicating that the phenomenon is related to a general abnormal behaviour of ASM and not to upregulation of specific agonist receptors (Curry, 1946; Dubois and Dautrebande, 1958; Nadel et al., 1965; Mcneill and Ingram, 1966; Mathé et al., 1973; Peters-Golden and Henderson, 2007; Driessen et al., 2012; Pascoe et al., 2012; Hyrkäs-Palmu et al., 2018). Another observation that points towards abnormalities in ASM is that a deep inhalation does not cause bronchodilation in asthmatic subjects, and in severe disease may lead to bronchoconstriction (Fish et al., 1981). This finding highlights the importance of stretch as a bronchorelaxant under normal circumstances. Furthermore, when non-asthmatic subjects are prevented from taking deep breaths, they experience AHR similar to asthmatic patients (Skloot et al., 1995). Thus, ASM stretching *via* breathing decreases airway resistance in normal lungs but not in asthmatic patients (Fish et al., 1981; Skloot et al., 1995).

While AHR has become accepted as a defining feature of asthma, the question of how each airway tissue component contributes to this phenomenon remains unanswered. Airway responsiveness measurements performed *in vivo* are likely to assess a combination of factors that include ASM contraction, airway wall stiffness and airway-parenchymal interdependence. As the constrictor of the airways, exaggerated ASM contraction is directly linked to AHR. Changes to ASM contractile properties and increases in ASM mass are likely the major players in enhanced airway responsiveness by causing airways to contract more easily in response to stimuli, often leaving the airways in a chronically contracted state (Pascoe et al., 2012; Berair et al., 2013). Increases in airway resistance in response to inhaled histamine is sensitive to the lung volume at which the measurements are made (Balassy et al., 1995). Higher lung volume reduces airway responses whereas at low volume, airway narrowing is exaggerated. These observations highlight the importance of mechanical impedances such as lung elasticity in determining the degree of airway narrowing for any particular activation state of the ASM. Given all these factors that contribute to AHR, elucidating the precise pathophysiology underlying exaggerated ASM shortening in asthma has not been easy.

Due to limitations in accessing and working with viable ASM tissue from human asthmatic subjects, a variety of animal models have been used to understand the role of ASM in asthma. These include the Fisher and Lewis rat model of innate AHR (Eidelman et al., 1991; Tao et al., 1999; Tao et al., 2003; An et al., 2006; Gil et al., 2006; Tao et al., 2012), horses with heaves which serves as a model of naturally occurring inflammatory asthma (Lowell, 1990; Leclere et al., 2011; Matusovsky et al., 2016), the Brown Norway rat and canine models of induced AHR (Antonissen et al., 1979; Gunst, 1983; Shore et al., 1983; Jiang et al., 1992; Watanabe et al., 1995; Ramos-Barbón et al., 2005; Matusovsky et al., 2014), and AHR following challenge of naturally sensitized sheep (Soler et al., 1991).

## Smooth muscle in the airway tree

The anatomy of the ASM differs based on its location in the airways (Suarez et al., 2012; Widmaier et al., 2019). Along the posterior end of the trachea and main bronchi, a transverse sheet of ASM connects the U-shaped cartilage rings of these segments. In the medium-sized bronchial generations, the ASM lies between the cartilage plaques and the epithelium. It extends to surround the entire airway so that its contraction leads to a decrease in airway lumen. In the next airway generations, the muscle strands become interwoven as a network of spirals that forms a mesh that lies between the cartilage and epithelium. The muscle strands become thinner as the caliber of the tubes becomes progressively smaller. In the bronchioles, the smooth muscle lies between the adventitial tissue and the epithelium but has become relatively large in proportion to the airway wall area, suggesting that it may have more functional importance in this airway generation (Macklin, 1929; Shore, 1984). Contraction of these spirally distributed ASM strands leads to a decrease in both airway diameter and length, which leads to a complex effect on airway resistance (Bates and Martin, 1990). Thus, contraction of ASM and the consequent bronchoconstriction compromises ventilation, although it may have a functional role in fine -tuning ventilation to perfusion ratios within the normal lung. While the role of ASM in healthy adult lungs remains unclear, recent findings have shown that ASM function extends beyond its ability to regulate bronchomotor tone (Camoretti-Mercado et al., 2021). For example, ASM cells can also have secretory or immunomodulatory functions (Ozier et al., 2011; Xia et al., 2013).

## Airway remodelling

Airway remodeling in asthma describes the changes that occur to structural components of the airway wall including changes in number, composition, distribution, thickness, and mass or volume (Fehrenbach et al., 2017). These changes arise in response to either a disturbance in lung development or chronic injury and/or lung inflammation (Fehrenbach et al., 2017). Remodeling has been observed to occur in various airway components including the epithelium, nerve tissue, bronchial vasculature and smooth muscle (Fehrenbach et al., 2017). Physical changes to the ASM, such as hypertrophy (increase in ASM cell size) and/or hyperplasia (increase in ASM cell number), are common in airway remodeling and which lead to increased ASM mass and therefore increased airway wall thickness (Cockcroft and Davis, 2006). Increases in ASM mass have been found to be associated with a decrease in lung function in severe asthma (Pepe et al., 2005; Kaminska et al., 2009). However, while the quantity of ASM was found to serve as an important factor contributing to differences in airway responsiveness between Fisher and Lewis rats, it could not account for variability of AHR within strain, suggesting that other factors relating to the ASM itself may impact AHR (Eidelman et al., 1991).

The extent of airway remodeling can be clinically correlated to asthma severity, as well as AHR (Boulet et al., 1997). Although ASM cells are structural in nature, they have also been shown to exhibit immunomodulatory functions and may secrete cytokines and chemokines, and express cellular adhesion molecules, which can be important for modulating airway inflammation (Panettieri, 2002). While it is unclear whether remodeling is driven by inflammation or if the two processes occur simultaneously, there is evidence to suggest that ASM itself may interact with the inflammatory cells and mediators in its surrounding environment in a positive feedback loop that furthers airway remodeling throughout asthma pathogenesis (Panettieri, 2002).

ASM from bronchial biopsies of asthmatic subjects has been shown to express CXCL10 and ASM cells stimulated with pro-inflammatory cytokines secrete CX3CL1 (Sukkar et al., 2004; Brightling et al., 2012; Manuel Tunon-de-Lara Ousova et al., 2022). These molecules are chemo-attractants for mast cells that infiltrate ASM and have the potential to alter its responses (Sukkar et al., 2004; Brightling et al., 2012; Manuel Tunon-de-Lara Ousova et al., 2022). Activation and degranulation of mast cells are known to play a role in the extracellular deposition of inflammatory products which may enhance ASM mass (Bara et al., 2010). Additionally, ASM can release a range of extra-cellular matrix (ECM) proteins such as fibronectin, perlecan, elastin, laminin, thrombospondin, chondroitin sulfate, collagen I, III, IV, and V, versican, and decorin (Johnson et al., 2012). The increased deposition of ECM proteins around the ASM layer can lead to fibrosis, which contributes to the thickening of the airway wall. The ECM proteins may also directly alter ASM function by enhancing its proliferation, such as is the case for fibronectin (Hirst et al., 2012). Another consequence of an increased ECM is the release of active transforming growth factor (TGFβ), which is stored in the ECM and can be cleaved by matrix metalloproteinases such as MMP-9 (Bara et al., 2010). This profibrotic cytokine enhances α-smooth muscle actin, myosin light chain kinase (MLCK), and smooth muscle myosin heavy-chain contractile protein expression in human ASM cells, impairs β_2_-agonist induced relaxation (Ojiaku et al., 2019), and may also increase ASM force of contraction and shortening in response to contractile agonists (Goldsmith et al., 2006). Another fundamental feature of asthma is the development of a strong effector T lymphocyte helper type 2 (Th2) response by the adaptive immune system (Murdoch and Lloyd, 2010). Th2 differentiated CD4^+^ T cells have been observed to form direct contacts with ASM in ovalbumin-sensitized Brown Norway rats where they induce DNA synthesis, cell proliferation, reduced ASM cell apoptosis, and increased ASM mass after ovalbumin challenge (Ramos-Barbón et al., 2005). In the same model, direct contact with ASM also decreased apoptosis of CD4^+^ T cells, thus suggesting a reciprocal cycle of remodelling driven by both the T cells and ASMCs.

Increased ASM mass is a hallmark of airway remodeling in asthma (Bara et al., 2010). It is characterized by both hypertrophy and hyperplasia, although the degree to which each occurs is not consistent across asthmatics (Bentley and Hershenson, 2008). Hypertrophy was found to be present in some severe asthmatics, and is suggested to be activated through two potential pathways, either involving the mammalian target of rapamycin (mTOR) or an inhibition of glycogen synthase kinase (GSK)-3β with Akt, also known as protein kinase B (Bara et al., 2010). mTOR phosphorylates eukaryotic initiation factor-4E (eIF4E)-binding protein (4E-BP) to release eIF4E, which plays a key role in the initiation of translation (Zhou et al., 2005). Increased 4E-BP phosphorylation leads to greater rates of protein translation and cell hypertrophy (Zhou et al., 2005). GSK-3β inactivates eIF2B, which also catalyzes a key regulatory step in mRNA translation (Welsh et al., 1998). Thus, the inhibition of GSK-3β may induce hypertrophy in an eIF2B dependent manner. Hyperplasia is induced by growth factors such as TGFβ, epidermal growth factor (EGF), and platelet derived growth factor, as well as stimulation by contractile agonists such as histamine and leukotriene D_4_ (Doeing and Solway, 2013). Reactive oxygen species and mechanical stress have also been implicated as factors that induce ASM cell proliferation (Bara et al., 2010). Indeed, it has been shown that bronchoconstriction *per se* can lead to EGF receptor ligand release (Tschumperlin et al., 2004) and ASM remodeling.

Whether it be through increased ASM mass or airway wall thickness, airway remodeling tends to leave ASM in a shortened state. Due to its ability to adapt its optimal length, this allows for airways to contract with greater force at smaller diameters, and also reduces the effectiveness of breathing and deep inspiration for dilating the airways in asthmatics (Seow and Solway, 2011; Chin et al., 2012). A positive feedback cycle has also been recognized in which ASM that has become too great in mass or receives too little opposing force will stiffen and stretch less, which will in turn lead to further stiffening (Oliver et al., 2007).

## Calcium release and myosin activation

ASM is innervated by the autonomic parasympathetic nervous system (Gosens et al., 2006). Activation of ASM is mediated by the neurotransmitter acetylcholine (ACh), which is released from nerve endings and binds to M3 muscarinic receptors, leading to an increase in cytoplasmic Ca^2+^ from the sarcoplasmic reticulum (SR) and the extracellular space, which is a key step in smooth muscle activation (Jude et al., 2008). Aside from activation through the nervous system, endogenous agonists that stimulate Ca^2+^ release include autocoids such as histamine and serotonin, lipid mediators such as cysteinyl leukotrienes C_4_, D_4_, and E_4_, prostanoids such as prostaglandins D_2_, F_2α_, and thromboxane A_2_ (Lam et al., 2019). Note that histamine also stimulates the release of Ach from nerve endings (Shore et al., 1983). Methacholine and carbachol are two drugs that mimic but are more stable *in vivo* than ACh so are used to study bronchoconstriction (Dubois and Dautrebande, 1958; Goldie et al., 1986; Gosens et al., 2006).

Under resting conditions, ASM cells maintain a low intracellular concentration of Ca^2+^. Contractile agonists stimulate G-protein-coupled receptors (GPCR) coupled to the Gα subunit and phospholipase C (PLC). Upon stimulation Ca^2+^ channels are opened throughout the cell to facilitate the influx of Ca^2+^ from the SR and the extracellular space (Jude et al., 2008; Perez-Zoghbi et al., 2009; Seow, 2021). The sarcolemma contains voltage-gated, Ca^2+^ store-operated, and receptor activated Ca^2+^ channels while the SR contains inositol 1,4,5-trisphosphate (IP_3_)-gated channels and ryanodine receptors (RyR’s) (Perez-Zoghbi et al., 2009; Seow, 2021). In addition, stimulation of the GPCR activates PLC which produces secondary messengers–inositol 1,4,5-trisphosphate (IP_3_) and diacylglycerol (DAG)—from the plasma membrane component phosphatidylinositol 4,5-bisphosphate (PIP_2_). IP_3_ releases Ca^2+^ from the SR, *via* the IP_3_-gated channels. Note that the Ca^2+^ increase in the cell is not monotonic but shows rhythmic oscillations (Perez-Zoghbi et al., 2009). These increases often start at one end of the cell and move throughout it as a wave, but the initiation site may fluctuate (Perez-Zoghbi et al., 2009).

**FIGURE 2:**
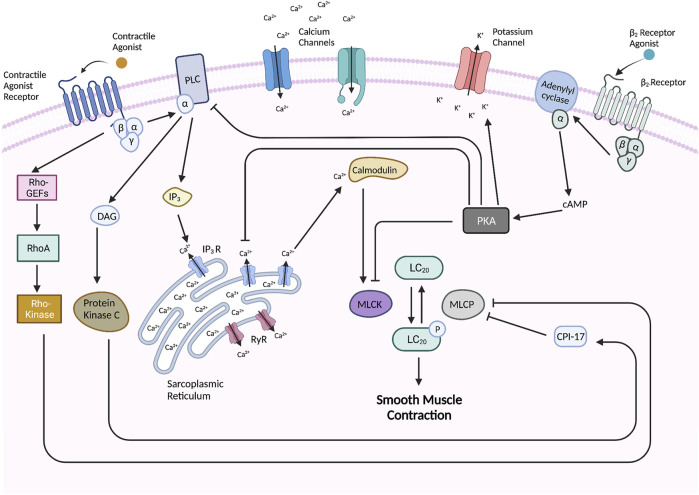
Molecular pathways underlying ASM contraction and relaxation. The activation of G-coupled protein receptors such as muscarinic and histamine receptors leads to subsequent interaction and activation of phospholipase C (PLC) which promotes the hydrolysis of phosphatidylinositol 4,5-bisphosphate (PIP_2_) into inositol 1,4,5-trisphosphate (IP_3_) and diacylglycerol (DAG). IP_3_ interacts with IP_3_-gated channels on the SR to induce Ca^2+^ efflux into the cytoplasmic space. Ryanodine receptors (RyR’s) on the SR also become activated to release Ca^2+^. In the cytoplasmic space, Ca^2+^ binds to calmodulin (CaM) which activates it so that it may activate myosin light chain kinase (MLCK) which phosphorylates and activates the myosin regulatory light chain (LC_20_) of myosin to initiate cross-bridge cycling and thus muscle contraction. LC_20_ is dephosphorylated and thus inhibited by myosin light chain phosphatase (MLCP), but this dephosphorylation is inhibited by two pathways–including the Rho-kinase pathway which is stimulated by the activated GPCR and a CPI-17 pathway which is activated by protein kinase C. Muscle relaxation can be induced by stimulation of a β_2_-receptor which leads to the activation of adenylyl cyclase (AC) which catalyzes the formation of cyclic AMP (cAMP). cAMP binds and activates PKA which carries out various regulatory activities such as inhibiting MLCK, PLC, and IP_3_-gated channels, and activating Ca^2+^-gated potassium channels in the cellular membrane to release K^+^ which hyperpolarizes the cell. Created with BioRender.com.

In the cytoplasmic space, Ca^2+^ binds to the protein calmodulin (CaM). The Ca^2+^-CaM complex then binds MLCK and activates it so that it can phosphorylate the myosin regulatory light chain (LC_20_). Once LC_20_ is phosphorylated, the myosin head is able to interact with actin filaments to generate force i.e., muscle contraction (Jude et al., 2008; Seow, 2021). Myosin light chain phosphatase (MLCP) dephosphorylates LC_20_, and thus mediates muscle relaxation (Seow, 2021). However, two parallel pathways can inhibit this dephosphorylation. A RhoA/Rho-kinase pathway, where Rho kinase, a serine/threonine kinase, phosphorylates and therefore inhibits MLCP (Somlyo and Somlyo, 2003; Chiba and Misawa, 2004). A second mechanism involves the inhibition of MLCP by CPI-17 that is activated when phosphorylated through a DAG/protein kinase C mechanism (Kitazawa et al., 2000). These two mechanisms are Ca^2+^ independent but enhance the effect of Ca^2+^ and have therefore been named Ca^2+^ sensitization. These Ca^2+^ dependent and independent activation mechanisms are depicted in Figure 2.

After an excitatory event, Ca^2+^ is taken up into the SR stores or pumped back into the extracellular space. The main Ca^2+^ storage compartment in ASM is the SR (Sanders, 2001). It is surrounded by a non-Ca^2+^ permeable membrane which is lined with specialized active Ca^2+^ ATPases known as SERCA (sarco/endoplasmic reticulum Ca^2+^-ATPase) pumps that maintain a 10,000-fold Ca^2+^ gradient between the SR lumen and the cytoplasm (Sanders, 2001). Mitochondria also play a role in Ca^2+^ uptake by way of a Ca^2+^ uniporter on the mitochondrial inner membrane that relies on an electrochemical gradient generated by the electron transport chain (Sanders, 2001). A major source of Ca^2+^ extrusion from the cytosol is the plasma membrane Ca^2+^-ATPase which uses ATP to transport Ca^2+^ back out of the cell in exchange for two protons. This exchange results in a net increase in protons within the cell which is compensated by transporters such as the Na^+^/H^+^ exchange (Sanders, 2001).

## Signalling pathways

The pathways leading to ASM contraction are complex so there are several potential steps that can be altered and lead to hypercontractility. Neural control has been shown through animal models to be implicated in AHR (Shore et al., 1985; Wagner and Jacoby, 1999; Lourenço et al., 2020; Nie et al., 2020) but a similar connection has not been proven in humans (Sterk et al., 1985). Thus, the focus on possible mechanisms to explain abnormal ASM behaviour remains on post-synaptic processes such as the interaction of acetylcholine and M_3_ receptors at the neuromuscular junction (Ouedraogo and Roux, 2003) and following pathways that become activated downstream.

Abnormalities in one or more of the signalling or contractile proteins can occur genetically or by alterations at the transcriptional, translational, or post-translational levels. A greater expression of the [(+) insert] smooth muscle myosin heavy chain (SMMHC) isoform–which is more highly expressed in rapidly contracting phasic muscle–has been hypothesized to contribute to AHR (Gil et al., 2006). The trachealis of the hyperresponsive Fisher rats was reported to express greater mRNA levels of the [(+) insert] SMMHC isoform and greater protein content of this isoform relative to total SMMHC compared to the normoresponsive Lewis rats (Gil et al., 2006). The asthmatic horses were also found to express higher (+) insert SMMHC at the mRNA and protein levels in intrapulmonary and central airways, which was reduced after corticosteroid treatment or antigen avoidance (Boivin et al., 2014). The (+) insert SMMHC isoform was also shown to be increased at the mRNA level in human endobronchial biopsies of well characterized asthmatic patients compared to controls (Léguillette et al., 2009). However, such studies have yet to be repeated at the protein level. Increased ASM MLCK protein levels have been reported in allergen sensitized human airways (Ammit et al., 2012), and a heightened MLCK mRNA expression was noted in asthmatic ASM cells (Ma et al., 2002; Léguillette et al., 2009). Similar results have been obtained from biopsies in asthmatics with differing levels of asthma severity (Zweiman et al., 2003). A higher MLCK content should increase the extent of LC_20_ activation, which has been correlated with a potentiated rate and extent of ASM shortening in the canine model of allergic AHR (Jiang et al., 1995). Heightened LC_20_ activation was observed in ASM from Fisher rats and in tracheal tissue from an allergic dog model of asthma (Jiang et al., 1995; Gil et al., 2006).

Another possible culprit of ASM hypercontractility is the dysregulation of Ca^2+^ homeostasis. Studies performed with human tissues reported a decreased expression of SERCA in asthmatic ASM (Mahn et al., 2009) whereas an enhanced Ca^2+^ release in response to contractile agonists was reported in the ASM of hyperresponsive Fisher rats (Tao et al., 1999). When the expression of this Ca^2+^ reuptake protein in healthy ASM culture was inhibited with siRNA, the cells exhibited asthmatic ASM behaviour such as increased motility, secretion of pro-inflammatory chemokines, and decreased Ca^2+^ reuptake (Tao et al., 1999). Therapies targeting the Ca^2+^ sensitization pathways have also been investigated and have revealed several promising candidates (Koziol-White et al., 2016; Yoo, 2017; Yoo et al., 2017).

ASM may also become hypercontractile due to abnormalities in the relaxation pathway. ASM relaxation can be induced by stimulation of the β_2_-receptors, which are GPCRs classified under the Gs sub-family (Billington and Penn, 2003). Upon activation, the G_s_ protein binds to and activates adenylyl cyclase which then catalyzes the formation of cyclic adenosine 3′,5′-monophosphate (cAMP) from cytosolic ATP (Billington and Penn, 2003). cAMP then binds with high affinity to the regulatory subunits of protein kinase A (PKA) and causes these subunits to dissociate from the enzyme complex (Sassone-Corsi, 2012). The remaining catalytic subunits of PKA can then phosphorylate and regulate the activity of numerous downstream proteins (Billington and Penn, 2003). Of note, PKA phosphorylates myosin light chain kinase (MLCK) which prevents downstream phosphorylation of myosin light chain (LC_20_) and thus promotes ASM relaxation (Billington and Penn, 2003). Activated PKA also phosphorylates other specific GPCRs, phospholipase C (PLC), and inositol 1,4,5-triphosphate (IP_3_) receptor which leads to a reduction of calcium flux (Billington and Penn, 2003; Álvarez-Santos et al., 2020). Another important effector of PKA are Ca^2+^ gated potassium (K_ca_) channels which are densely distributed throughout the ASM cellular membrane (Kume et al., 1994). Upon phosphorylation, these channels open and allow K^+^ efflux which results in cell hyperpolarization. Figure 2 depicts the β_2_-mediated relaxation pathway.

Many inflammatory cytokines present in asthmatic airways decrease the relaxant response to isoproterenol in human ASM, including interleukin (IL)-13, IL-1β, tumour necrosis factor (TNF), and TGF-β through several different mechanisms (Shore, 2002). IL-13 decreases the β_2_-adrenergic response *via* an extracellular signal-regulated kinase (ERK) dependant pathway (Laporte et al., 2001) whereas IL-1β & TNF induce the expression of cyclooxygenase-2, resulting in prostanoid release (Laporte et al., 1998). IL-13 and TNF also augment Ca^2+^ release in response to contractile agonists, and IL-13 increases the expression of receptors for histamine and cysteinyl leukotrienes (Amrani et al., 1997; Deshpande et al., 2004; Manson et al., 2020). Additionally, IL-13 has been shown to increase ASM constriction in response to acetylcholine and histamine (Grunstein et al., 2002; Risse et al., 2011).

## Methods to measure airway smooth muscle mechanics and outcomes

### Excised airway segments

Excised airway segments from animal models or post-mortem human lungs have been used to study airway mechanics and to infer ASM mechanics. The side branches of the airway segments are sealed off to form a leak-free system, allowing measurements of ASM force generation through changes in pressure or volume (Gunst and Mitzner, 1981; Noble et al., 2007). When using airways from small animals, this can be done by measuring the pressure drop of a fluid perfused along an airway segment. When using airways from large animals *ex vivo*, it is possible to directly study the lumen of the airways either by endoscopy, anatomical optical coherence tomography, or ultrasound. While the excised segments retain many elements of the airways including the epithelial layer, nerves, and airway wall, they also provide an opportunity to perform more intrusive protocols such as electrical field stimulation and to compare differences between the central and peripheral airways (LaPrad et al., 2008; LaPrad et al., 2010; Noble et al., 2010; Ansell et al., 2013). The dynamics of stretch as a bronchodilator have been studied using excised airway segments (LaPrad et al., 2010; Ansell et al., 2013). It has been shown that applying strain rather than stress induces bronchodilation (Ansell et al., 2013). Furthermore, rapid stretches cause greater bronchodilation (Ansell et al., 2013) than slow ones (Ansell et al., 2013). One drawback to the approach of a fully integrated system in airway models is that the elucidation of cellular and molecular mechanisms is not possible, and thus studies using these models are often supplemented with *in vitro* experiments such as the use of human ASM cell culture.

### Precision cut lung slices

*In vitro* experiments using human lungs are limited by the relative inaccessibility of smaller intrapulmonary airways, making it difficult to study the airways in the more peripheral regions (Lam et al., 2019). The early thick slices (Dandurand et al., 1993) have given way to thinner precision cut lung slices (PCLS) (Martin et al., 1996; Perez-Zoghbi et al., 2009; Wright et al., 2013). PCLS offer an approach to study ASM *ex vivo* in an integrated system (Martin et al., 1996; Wright et al., 2013; Lam et al., 2019). PCLS are prepared by filling intact lungs, lobes, or wedge preparations with liquid agarose that maintains tissue structure after solidifying (Martin et al., 1996; Lam et al., 2019; Liu et al., 2019). After the gel stiffens, it is then possible to generate lung slices that retain many features of the airway anatomy including the alveolar architecture, respiratory parenchyma, structural and immune cells, and connective tissue. PCLS can be maintained in culture medium for up to 14 days, where they maintain normal metabolic activity, tissue homeostasis, and structural integrity (Liu et al., 2019). As PCLS can be maintained for extended periods of time, it is also possible to study the impact of prolonged exposures to various stimuli (Cooper and Panettieri, 2008; Cooper et al., 2011). Innate immune responses have been observed in PCLS after lipopolysaccharide exposure, bacterial infections, or viral infections (Chakrabarty et al., 2007; Henjakovic et al., 2008; Wu et al., 2010). The presence of other airway cells in PCLS also allows the assessment of the effects of airway inflammation and remodelling on ASM contraction.

Bronchoconstriction in response to agonists and electric field stimulation (EFS) can be captured and measured in real-time with videomicroscopy and digital imaging (Martin et al., 1996). To perform this method, PCLS are held down on a cover slip by a nylon mesh which has a hole cut in the middle to expose the airway (Bai and Sanderson, 2009). A second cover slip is placed over the mesh and sealed with silicon grease to create a perfusion chamber, and the lung slice is perfused by a gravity driven perfusion system (Bai and Sanderson, 2009). PCLS retains the parenchymal load against the contracting ASM, despite a loss of the 3D structure (Dandurand et al., 1993; Wright et al., 2013; Lam et al., 2019) so that experiments can be carried out without setting and controlling an arbitrary ASM length and tension, reducing the complexity of the measurements. The early studies using lung slices to compare inbred strains of rat differing in airway responsiveness showed that the intraparenchymal airways of the hyperresponsive Fisher rats constricted faster and to a larger extent than those of the normoresponsive Lewis rats, as had previously been reported *in vivo* (Tao et al., 1999). The AHR was also manifest at the ASM cellular level; Ca^2+^ mobilization by an agonist was enhanced in the Fisher compared to the Lewis rat (Tao et al., 2003; Tao et al., 2012).

PCLS has been a valuable tool for the study of Ca^2+^ signalling while retaining the gross architecture. ASM can also be loaded with fluorescent dyes to detect Ca^2+^ flux and sensitivity (Liu et al., 2019). PCLS have also been used to study Ca^2+^ oscillations in ASM and towards understanding the contributions of various Ca^2+^ channels (Perez-Zoghbi et al., 2009). The PCLS technique has been used with disease models as well as with transgenic mice to dissect the contributions of highly specific phenotypes (Sanderson, 2011). Through a combination of mathematical modelling and experimental work involving Ca^2+^ channel inhibitors, store-operated Ca^2+^ channels were shown to play a prominent role in maintaining Ca^2+^ and thus revealed their critical importance for sustaining ASM contraction and tone (Boie et al., 2017; Chen and Sanderson, 2017). Although not having as significant a role in replenishing Ca^2+^ during ASM contraction, PCLS studies in mice also have shown that the level of RyR sensitization impacts Ca^2+^ oscillations. Agonist-induced contractions result in Ca^2+^ depletion of the SR which normally causes RyR inactivation (Croisier et al., 2015). However, inflammatory cytokines such as TNF-α and IL-13 have been reported to modify RyR, possibly causing them to remain open during SR depletion, increasing Ca^2+^ sensitivity (Deshpande et al., 2004; Sieck et al., 2008). Small increases in the sensitivity of cytosolic and/or luminal RyR led to low-frequency Ca^2+^ oscillations while moderate to high luminal RyR sensitization led to an elevated average cytosolic Ca^2+^ concentration upon agonist stimulation (Croisier et al., 2015).

One important point to consider when using PCLS is the loss of diffusion barriers that are normally present in the 3D structure of the airway because agonists or mediators added to the culture can access all compartments of the airways. Without the epithelial barrier, the ASM is now directly exposed to various stimuli (Wright et al., 2013). This makes it difficult to observe which cell type is being affected by the stimuli and determine the exact source of a secreted endogenous substance. PCLS will also lose certain cell populations during longer-term culture, which can contribute to decreased sensitivity in response to external stimuli (Liu et al., 2019). However, insulin has been shown to preserve myosin heavy chain expression in mice PCLS when maintained in culture for 2 weeks and retains IL-13-induced ASM hypercontractility (Li et al., 2020).

PCLS used with post-mortem human lungs is improving our understanding of AHR. With PCLS, a reduced airway relaxation to isoproterenol was observed in the presence of ASM β_2_ receptor phosphorylation (Tyr 141 and Tyr 350), an effect that is induced in asthma by growth factors released from activated mast cells (Chachi et al., 2018). The PCLS technique has also been used to demonstrate the dilation of small airways by PI3k inhibitors (Koziol-White et al., 2016) and to study the potentiation of ASM contractility by inflammatory cytokines such as IL-17 (Willis et al., 2015). However, thus far there are no reports showing intrinsic differences in agonist responses of ASM between human asthmatic and control PCLS.

### Airway smooth muscle strips

Although there are many benefits to measuring ASM contraction in integrated systems, one drawback to models using airway segments and PCLS is that the ASM contractile properties cannot be isolated from the influence of the other tissue elements. The presence of multiple cell types and a complex mechanical environment impose potential modulating effects which may mask the behaviour of ASM (Gunst, 2012). Therefore, the practice of using isolated ASM strips or bronchial/tracheal ASM rings has been employed to fill this knowledge gap. ASM strips have been obtained from animal species such as mice, rats, guinea pigs, dogs, pigs, horse, and cows, but human ASM strips are also used and obviously, provide the most relevant information for understanding asthma (Gunst, 1983; Wang et al., 2000; Latourelle et al., 2002; Moir et al., 2003; Bullimore et al., 2011; Dekkers et al., 2012; Matusovsky et al., 2016). Using ASM from larger animals such as horses has been integral to obtaining initial data regarding the deeper generations of the airways (Matusovsky et al., 2016). Due to limited supply of asthmatic human ASM tissue, control human ASM has been sensitized through incubation with asthmatic serum, IgE, or cytokines including IL-13, IL-5, TNF, and IL-1β to mimic the asthmatic inflammatory milieu (Black et al., 1989; Ellis et al., 1994; Tunon de Lara et al., 1995; Barchasz et al., 1999; Hakonarson et al., 1999; Grunstein et al., 2002).

ASM can be dissected from isolated airways, leaving a tissue strip, with or without epithelium, with muscle fibres that are aligned along the longitudinal axis. The strips can be attached to two aluminum foil clips which are used to mount the ASM on a force transducer that senses changes in contractile force and a length-sensing lever (Chin et al., 2012). This system allows the control over two important variables that characterize ASM function–force and length. It is then possible to focus on each variable independently of one another by studying either isometric or isotonic contractions. Changes in tissue tension (i.e., force) are measured by exposing the strip to contractile or relaxing agonists, or an electric field stimulation (EFS) (Ijpma et al., 2015). Assessing tissue changes in tensile force in response to gradually increasing concentrations of a drug or agonist gives a dose-response curve that has been used to compare differences in ASM responsiveness (see above) (O’Byrne and Inman, 2003). Using the quick-release technique (described below), it is possible to determine the shortening velocity (Seow and Stephens, 1986; Bullimore et al., 2010; Ijpma et al., 2015).

Important parameters of muscle mechanics include force, stress, length, and shortening velocity. Smooth muscle differs from striated muscle in that it is plastic and adapts to changes in length. It has, as for striated muscle, an optimal length at which it produces maximal isometric force (Stephens and Van Niekerk, 1977). However, SM changes its internal configuration to adapt to changes in length quite quickly. It is therefore important to maintain a constant reference length throughout testing conditions. Thus, changes in ASM length during a contraction are expressed as a percentage change of a predefined reference length (Chin et al., 2012; Matusovsky et al., 2016; Luo et al., 2019).

The magnitude of force generated by a smooth muscle bundle depends on the number of activated cross-bridges (Seow, 2021). Thus, an important parameter is the maximal force, obtained with maximal stimulation, and which is designated as F_max_ (Matusovsky et al., 2016; Seow, 2021). Under *in vitro* conditions, this is usually measured at a fixed length, as the maximal isometric force. Muscles and cell bundles with larger cross-sectional areas can generate more contractile force because they have more myosin molecules that work in parallel. This is important to note as an increased ASM mass is a common feature in asthmatic airways (Dunnill et al., 1969; Carroll et al., 1993). Note that this increased ASM mass has been examined by stereology and was believed to be overestimated due to matrix proteins between ASM cells (Thomson et al., 1996). However, further careful stereology studies did not confirm any difference in matrix proteins between asthmatic and control ASM (James et al., 2012). Thus, to study ASM responses that are otherwise influenced by ASM mass, muscle force is normalized to the cross-sectional area which is referred to as stress (Ijpma et al., 2015).

$$Stress=\frac{Force}{Muscle\;cross\;sectional\;area}$$

The velocity of muscle shortening describes the rate at which the length of a muscle decreases during a contraction (Chin et al., 2012; Seow, 2021). ASM shortening velocity is inversely related to the load against which a muscle contracts. Velocity is highest in the initial stage of contraction and decreases over time throughout muscle shortening. Thus, for comparative purposes, it is important to measure velocity at the same timepoint after muscle stimulation.

Force-velocity plots are constructed using the quick-release technique. Briefly, the muscle is first activated under isometric conditions until the force generated reaches a plateau (Seow, 2021). At this point, the contraction is switched from isometric to isotonic (at a given specific load), which allows muscle shortening from its initial length. Assuming no viscoelastic recoil, the muscle’s shortening velocity at this load is taken as the change in length per unit of time. Repeating this with progressively declining loads generates the data that form a force-velocity plot. In theory, the muscle would achieve its maximal velocity (V_max_) if the load were dropped to zero, thus V_max_ is calculated by extrapolation to the zero load of this plot. V_max_ is commonly used because it is a good representation of the actin-myosin cross-bridge cycle.

Historically, there has been ambiguity in the data addressing whether ASM function is abnormal in asthma. While some *in vitro* studies using asthmatic ASM have demonstrated increased force generation, shortening, and agonist sensitivity (Schellenberg and Foster, 1984; de Jongste et al., 1987; Bai, 1990; Bramley et al., 1994; Thomson et al., 1996), others have also shown no difference (Roberts et al., 1984; Goldie et al., 1986; Whicker et al., 1988; Cerrina et al., 1989; Bjorck et al., 1992). However, many of the earlier studies were performed with small sample sizes and with various methodological limitations such as the lack of normalization of force with respect to ASM cross-sectional area, or without the capacity of measuring ASM shortening and shortening velocity (Seow et al., 2012).

Later studies that collected more robust data reported significantly increased maximal contractile capacity and shortening velocity in response to EFS as well as an increased response from asthmatic ASM cells to contractile agonists, such as histamine and bradykinin (Ma et al., 2002; Matsumoto et al., 2007; Sutcliffe et al., 2012). More recent studies that compared the ASM function between asthmatics and normal controls, used isolated human ASM bundles from the trachealis and main bronchi and they did not report any difference in stress and Vmax (Chin et al., 2012; Ijpma et al., 2015). However, increased passive stress and altered response to length oscillations were seen (Chin et al., 2012; Ijpma et al., 2015). An equine model of spontaneous and severe asthma was then used to study the ASM mechanics of intrapulmonary (IP) airways. IP ASM of the asthmatic horses exhibited a greater V_max_ than their own trachealis, and greater than IP ASM of control horses, whereas the IP ASM of horses under remission showed increased stress (Matusovsky et al., 2016). Following the optimization of the IP ASM dissection done in the horse, comparisons between human IP (i.e., Third-fifth generations) and tracheal segments were performed and significantly greater contractile stress and stiffness were reported for the asthmatic IP ASM strips (Ijpma et al., 2020).

Modelling and ventilation imaging approaches have indicated heterogeneous airway constriction during asthmatic exacerbations with an emphasis on the importance of small airway constriction (Tgavalekos et al., 2005; Tzeng et al., 2009). These site -specific changes in ASM intrinsic contractility are speculated to be influenced by increased interactions with an inflammatory environment in the IP airways (Lutchen, 2016). The severity of inflammation has been shown to progressively increase towards the periphery (Hamid et al., 1997; Balzar et al., 2002; De Magalhães Simões et al., 2005), therefore IP ASM will encounter a greater number of inflammatory cells/mediators compared to that of the trachea and main bronchi. Of note, the increase in V_max_ seen from the equine model was dependent on the time since the last corticosteroid treatment, indicating that acute inflammation enhanced ASM mechanics (Matusovsky et al., 2016). Conversely, the increased IP ASM stress observed in the horses under remission is presumably due to long term ASM remodelling. The normal Vmax but increased stress reported in the human IP ASM by Ijpma et al. (2020) were obtained from asthmatic subjects who died of other causes than asthma, and so did not necessarily exhibit high levels of inflammation. Their ASM properties were therefore closer to those of the horses under remission. Taken together, these data suggest that mechanical alterations observed in asthmatic IP ASM, depend on the type and level of inflammation in the surrounding airway environment and on the chronicity of the exposure.

It is noteworthy that while we often think of a hypercontractile ASM as a stronger muscle, ASM that exhibits a greater Vmax could, conceivably, lead to AHR. It has been suggested that due to the stretching and relaxing effect of tidal breathing, an ASM that re-contracts rapidly between each breath would maintain asthmatic airways in a more constricted state because the ASM would have time to shorten significantly between each breath, thus counteracting the relaxing effect of tidal breathing (Gunst, 1983; Solway and Fredberg, 1997). Indeed, computed tomography and transfer impedance studies showed that both healthy and asthmatic airways dilate upon lung inflation, but that the asthmatic airways rapidly narrow back to their initial diameter (Brown et al., 2001; Jackson et al., 2004). In addition, a greater Vmax has been observed in sensitized human bronchi (Mitchell et al., 1994), in single ASM cells from asthmatic human airways (Ma et al., 2002), and in mouse, rat and canine models of AHR (Antonissen et al., 1979; Jiang and Stephens, 1990; Duguet et al., 2000; Blanc et al., 2003; Fan et al., 2012).

### Airway smooth muscle culture and airway smooth muscle cell mechanics

The development of primary human ASM cell culture has provided substantial information on the molecular pathways and cellular interactions that influence ASM contraction (Hirst, 1996). ASM cells can be cultured through two methods (Hirst, 1996). In the first, cells are enzymatically dissociated from a minced ASM preparation (Hall and Kotlikoff, 1995). With the second approach, cells are grown from explants of ASM in petri dishes or tissue-culture flasks and eventually harvested after growing to confluence (Hall et al., 1992; Marsh and Hill, 1992).

ASM cell contraction can be studied using various methods. One frequent, although indirect measurement, is that of the concentration of intracellular Ca^2+^, which increases when ASM cells are triggered to contract. To do so, cells are loaded with the fluorescent Ca^2+^ indicator Indo-1 and monitored fluorometrically with an Indo-1 probe (Berger et al., 2001). Ca^2+^ can also be quantified using the ratiometric fluorophore FURA2-AM. Measurements of downstream events including the phosphorylation of LC_20_ are also made using western blots (Hunter et al., 2003). For greater sensitivity in quantifying LC_20_ phosphorylation, western blots using Phos-tag SDS PAGE gels can increase protein solubility and extraction efficiency, and results are confirmed with phosphor-Ser19- LC_20_ antibodies (Takeya et al., 2008). Cell contraction can also be more directly measured using a collagen gel substrate embedded with ASM cells (Lee et al., 1995; Matsumoto et al., 2007; Han et al., 2020). When assessing contraction, the gels are released from the wells and a contractile stimulus is added. The decrease in gel area can then be taken as a measure of cell contraction. This method requires caution while interpreting results as the free-floating gel provides very little load opposing the ASM, thus allowing for non-contractile cytoskeletal re-organization to manifest as a contraction. Moreover, it is infeasible to “relax” the gel and obtain repeated measurements (Ceresa et al., 2009). Collagen gel contractions have shown that asthmatic ASM cells contract significantly more in response to histamine compared to control ASM cells (Matsumoto et al., 2007).

Cultured ASM cells stiffen when exposed to contractile agonists (An et al., 2002). The stiffening may be measured by optical magnetic twisting cytometry (Wang and Ingber, 1994; Fabry et al., 2001; An et al., 2006; Wright et al., 2013). To do this, ferromagnetic microbeads that are coated with a synthetic RGD (Arg-Gly-Asp)-containing peptide are bound to the cell cytoskeleton *via* integrin-receptors on the cell surface. The beads are first magnetized horizontally (parallel to the cell plating surface) with a strong homogenous pulse and then twisted vertically with the application of a weaker external homogeneous magnetic field that varies sinusoidally in time. This results in displacement of the bead which can be detected optically and can be used alongside the applied torque value to calculate the cell’s stiffness. Changes in stiffness can be detected upon subsequent additions of contractile or relaxant agonists and can be related back to the contractile force. Using this approach, An and co-workers showed that ASM cells from the hyperresponsive Fisher rat exhibited greater and faster stiffening responses, as well as greater contractile forces, than those of the Lewis rats (An et al., 2006). ASMCs from Fisher rats also demonstrated a faster rate of cytoskeletal remodeling, allowing for the ASM cell to adapt to progressively shorter working lengths which is thought to be a major factor in contributing to excessive narrowing (An et al., 2006). More recently, using this technique, An et al. also showed enhanced stiffness in cells harvested from asthmatic subjects (An et al., 2016).

Another technique to estimate ASM cell force production is traction force microscopy (TFM) (Wang and Li, 2009). TFM is primarily used to quantify forces that contribute to cell migration, but one can also obtain a scalar measure of contractile strength known as the net contractile moment (An et al., 2009). TFM employs an elastic polyacrylamide substrate that is coated with ECM proteins and embedded with fluorescent particles. ASM cells are plated on this substrate and displace the fluorescent particles during a period in which they spread and stabilize. The displacement of cells can then be microscopically tracked after the cells are removed *via* trypsinization (Wright et al., 2013). This technique has shown that asthmatic ASM cells exhibit increased cell traction forces at baseline and also demonstrate a higher net contractile moment compared to non-asthmatic cells (An et al., 2016).

### Novel therapeutics for asthma and potential effects on airway smooth muscle

Following the recognition that several asthma phenotypes exist and that they impact the response to treatments, there has been great interest in developing biological therapies to target specific immune cells and inflammatory mediators (Zhu et al., 2018). These include antibodies against IL-13, IL-4 receptor α, CRTHT2, TSLP, IL-25, IL-13, IL-17A receptor, and CXC chemokine receptor 2 (CSCR2/IL-8) (Zhu et al., 2018). Certain mediators such as IL-4, IL-13, IL-25, and IL-17A are known to cause ASM hyperplasia and AHR (Kudo et al., 2012; Willis et al., 2015; Robinson et al., 2017), but most novel biologics are focused on their effects on inflammatory pathways. Of interest, IL-13 has been shown to enhance ASM contraction and impair relaxation (Corren, 2013). Dupilumab–an anti-IL-4/IL-13 biologic–was described to attenuate human ASM remodelling and contraction as assessed by studies using small bronchi explants and cell culture (Manson et al., 2020) and demonstrated efficacy in phase 3 trials (Wenzel et al., 2016; Robinson et al., 2017). IL-17A is another mediator that directly increases the contractility of ASM as was demonstrated in an allergen sensitized mouse model (Kudo et al., 2012). To inhibit the effects of IL-17A, brodalumab–which binds to the IL-17 receptor A and thus blocks IL-17A–was tested in a clinical trial with patients who had uncontrolled moderate to severe asthma, but did not demonstrate significant improvement in the overall study population, although a nominal significance in Asthma Control Questionnaire scores was observed in one treatment subgroup (Busse et al., 2013a). Current Food and Drug Administration-approved biologics such as tezepelumab which blocks TSLP (Menzies-Gow et al., 2021), omalizumab which blocks immunoglobulin (Ig)E (Busse et al., 2013b; Hanania et al., 2013; Chipps et al., 2017), and mepolizumab and reslizumab which bind and neutralize IL- 5 (Nair et al., 2009; Castro et al., 2012) were found to improve various asthmatic symptoms such as reducing exacerbation rates, free IgE levels, blood and sputum eosinophils and improved FEV_1_. However, their effects on ASM and AHR have not been identified. Bronchial thermoplasty is another novel treatment for adults with severe asthma that shows promising results (Dombret et al., 2014). With this technique, thermal energy is used to reduce the presence of ASM in the airways. The procedure is described to be minimally invasive, and patients in a 3 month follow-up have reported more symptom-free days, fewer exacerbations, and improved expiratory flow and airway responsiveness (Cox et al., 2007; Cox et al., 2012). However, a temporary worsening of respiratory symptoms was observed after the initial treatment period so there are concerns that the application of heat in the airways may inflict further injury on the bronchial wall (Dombret et al., 2014). Further studies are still required to explore the long-term effects of the procedure. As it is difficult to isolate ASM effects in patients, the benefit of many novel treatments on ASM remains unknown. As such, a greater understanding of ASM mechanics and its role in the airways will be beneficial for the identification and development of more targeted therapeutics.

## Conclusion

Asthma is a multifactorial disease with alterations at several levels including changes to ASM contractility. Such changes derive from factors including airway hyperresponsiveness (which affects ASM sensitivity and reactivity to contractile agonists and reduces β_2_-receptor mediated relaxation) and airway remodeling (which results in increases in ASM mass that worsens the effect of bronchoconstriction). Alterations in calcium flux and dysregulation in the expression of signalling or contractile proteins occur by alterations at the transcriptional, translational, or post-translational levels possibly caused by heightened interactions with inflammatory mediators in asthmatic airways. The role of ASM in asthma is depicted through the measurement of its intrinsic mechanical features and how they change throughout disease pathogenesis. Multiple approaches have been used to understand the abnormalities of ASM in asthma, with each presenting different advantages and limitations. *Ex vivo* airway segments and precision cut lung slices may provide measurements that account for the interactions between ASM and other airway components while the ASM strip models focus on resolving important parameters of ASM mechanics including stress, length, and shortening velocity. ASM cell mechanics illustrate changes at a cellular level and can also be useful for providing an understanding of the changes at a molecular level through demonstrating the modifications that occur to contractile proteins and receptors. Current treatments for asthma may alleviate symptoms but cannot serve as a cure. Due to asthma having multiple phenotypes where each presents different underlying drivers of disease, conventional treatments are still insufficient in many cases and only work for specific types of the disease. Notwithstanding the insights gained to date, the elucidation of the root cause of the abnormal *in vivo* behaviour of ASM remains an important goal because ASM is the downstream effector of airway obstruction and AHR. A better understanding of the adverse changes that happen to ASM and its role in asthmatic airways may allow the discovery of therapeutics that provide a permanent cure and improved quality of life for asthmatics.
